# Global Effects of Catecholamines on *Actinobacillus pleuropneumoniae* Gene Expression

**DOI:** 10.1371/journal.pone.0031121

**Published:** 2012-02-08

**Authors:** Lu Li, Zhuofei Xu, Yang Zhou, Lili Sun, Ziduo Liu, Huanchun Chen, Rui Zhou

**Affiliations:** Division of Animal Infectious Diseases in State Key Laboratory of Agricultural Microbiology, College of Veterinary Medicine, Huazhong Agricultural University, Wuhan, China; East Carolina University School of Medicine, United States of America

## Abstract

Bacteria can use mammalian hormones to modulate pathogenic processes that play essential roles in disease development. *Actinobacillus pleuropneumoniae* is an important porcine respiratory pathogen causing great economic losses in the pig industry globally. Stress is known to contribute to the outcome of *A. pleuropneumoniae* infection. To test whether *A. pleuropneumoniae* could respond to stress hormone catecholamines, gene expression profiles after epinephrine (Epi) and norepinephrine (NE) treatment were compared with those from untreated bacteria. The microarray results showed that 158 and 105 genes were differentially expressed in the presence of Epi and NE, respectively. These genes were assigned to various functional categories including many virulence factors. Only 18 genes were regulated by both hormones. These genes included *apxIA* (the ApxI toxin structural gene), *pgaB* (involved in biofilm formation), APL_0443 (an autotransporter adhesin) and genes encoding potential hormone receptors such as *tyrP2*, the *ygiY*-*ygiX* (*qseC*-*qseB*) operon and *narQ-narP* (involved in nitrate metabolism). Further investigations demonstrated that cytotoxic activity was enhanced by Epi but repressed by NE in accordance with *apxIA* gene expression changes. Biofilm formation was not affected by either of the two hormones despite *pgaB* expression being affected. Adhesion to host cells was induced by NE but not by Epi, suggesting that the hormones affect other putative adhesins in addition to APL_0443. This study revealed that *A. pleuropneumoniae* gene expression, including those encoding virulence factors, was altered in response to both catecholamines. The differential regulation of *A. pleuropneumoniae* gene expression by the two hormones suggests that this pathogen may have multiple responsive systems for the two catecholamines.

## Introduction

During infection, bacteria can actively respond to the mammalian hormones that are elevated significantly after stress [1]. This response provides an important bridge between infectious disease and stress, and lead to the concept of “microbial endocrinology” [2], [3], [4]. The catecholamines dopamine, epinephrine (Epi) and norepinephrine (NE), which are all derived from tyrosine, are widely distributed in animal tissues as stress related hormones and neurotransmitters. During stress, the hypothalamic-sympathetic system causes rapid release of Epi into the plasma from the adrenal medulla [5]. NE is released locally from peripheral sympathetic nerve endings and is also present in the blood stream following release from the adrenal medulla [5]. Recently, it was discovered that phagocytes (including macrophages, neutrophils and blood polymorphonuclear cells) synthesized and released catecholamines [6]. Microbial endocrinology studies have revealed that catecholamines not only play essential roles in stress and immune responses, but also trigger pathogen responses. One effect of catecholamines on bacteria is to stimulate growth by facilitating iron removal from host iron binding proteins [7], [8], [9]. Expression of other bacterial virulence factors is also affected by catecholomines. For example, in *Escherichia coli*, catecholamines can increase adhesion to host cells by up-regulating adherence associated genes [10], [11], [12], [13]. In *E. coli*, catecholamines can also affect chemotaxis, colonization to Hela cells [14] and enhance Shiga toxin production [15], [16]. Two adrenergic receptors, the sensor kinases QseC and QseE, have been identified in *E. coli* O157:H7 [17], [18], [19]. Many other species including respiratory pathogens can also respond to catecholamines [8], [20], [21].

*Actinobacillus pleuropneumoniae*, a member of the family *Pasteurellaceae*, is the etiologic agent of porcine contagious pleuropneumonia which causes great economic losses in the pig industry worldwide [22]. Many virulence factors have been reported in *A. pleuropneumoniae* including pore-forming exotoxins (ApxI-III) [23], capsular polysaccharide (CPS) [24], lipopolysaccharide (LPS) [24], protease [25], [26], urease [27], [28], iron-acquisition proteins [24] and enzymes involved in anaerobic respiration [29], [30]. Some putative adhesins (autotransporter adhesins [31], [32], type IV pilus [33], [34] and Flp pilus [35]) and those associated with biofilm formation [35], [36] are also suggested to be associated with infection.

Stress can increase susceptibility to infectious disease [37]. In swine herds, the incidence of respiratory diseases including pneumonia is often increased after stress [38]. Catecholamine production is an essential stress response of animals and high levels of catecholamines are related to acute respiratory illness [39]. In pigs, the plasma catecholamine levels can significantly increase after cold stress and lead to decrease of immunity [40]. A dense network of noradrenergic nerve fibres is present in the pulmonary tissue in pigs, consistent with observations that adrenergic components can influence pig lung function [41]. Therefore, the roles of catecholamines on the pig respiratory system after stress could be important. Stress factors such as crowding, transportation, moving of pigs and adverse climatic conditions contribute to *A. pleuropneumoniae* infection and transmission [42]. Following such stresses, the morbidity and mortality of disease are consequently affected [42], but the mechanism of this phenomenon is unknown. The swine respiratory pathogen *Mycoplasma hyopneumoniae* can actively respond to norepinephrine [21]. Another swine respiratory pathogen, *Bordetella bronchiseptica*, can use catecholamines to stimulate growth [8], [20]. These observations indicate that stress hormones can regulate the behavior of swine respiratory pathogens. The outcome of host-pathogen interaction may be affected. To discover whether *A. pleuropneumoniae* can respond to catecholamines, global analyses of *A. pleuropneumoniae* gene expression in response to Epi and NE were conducted by transcriptional profiling. Selected differentially expressed genes were analyzed and the influences of Epi and NE on cytotoxicity, biofilm formation and adhesion of this bacterium were further investigated.

## Materials and Methods

### Bacterial strain, cell line and culture conditions

*A. pleuropneumoniae* 4074 (serovar 1 reference strain) was cultured aerobically in Tryptic Soy Broth (TSB) (Difco Laboratories, USA) with rotation at 200 rpm or on Tryptic Soy Agar (TSA) (Difco Laboratories, USA) supplemented with 10 µg/ml of nicotinamide adenine dinucleotide (NAD) and 10% (v/v) filtered cattle serum at 37°C. When detecting the effects of catecholamines and/or their antagonists on *A. pleuropneumoniae*, Epi, NE, α-adrenergic receptor antagonist phentolamine (PE) and β- adrenergic receptor antagonist propranolol (PO) (Sigma, USA) were added to a final concentration of 50 µM [43]. The St. Jude Porcine Lung cell line (SJPL; kindly donated by Prof. Robert G. Webster, St. Jude Children's Research Hospital, USA) [35] was grown at 37°C in 5% CO_2_ in Dulbecco's modified Eagle's medium (DMEM; Gibco, USA) supplemented with 10% fetal bovine serum.

### Microarray construction

The microarrays used in this study consisted of 15744 60-mer oligonucleotide probes synthesized *in situ* by Agilent Technologies. The probes were designed based on the genome sequences of *A. pleuropneumoniae* 4074 (serovar 1), JL03 (serovar 3) and L20 (serovar 5) (GenBank accession numbers: AACK00000000, CP000687, CP000569 ) and included 2132 ORFs. Each probe with the same sequence for a given gene was repeated twice on the array.

### Sample preparations and microarray experiments

The Epi and NE treated and the untreated samples were collected from mid-log phase cultures. Three independent biological replicates were performed. Total RNA was extracted using RNA-Solv Reagent (Promega, USA) according to the manufacturer's instructions. Hybridization and scanning were conducted according to the Agilent microarray experiment protocols (Agilent, USA).

### Data analysis

The signal intensities were normalized using Feature Extraction Software (Agilent, USA) and transformed into log2 values. The genes with positive signals (flags = P or M) in all hybridizations were selected to be further analyzed. The genes with fold change ≥1.5 and *P*<0.05 using the software TM4 were selected as differentially expressed genes. As a result of poor annotation of the unfinished genome of *A. pleuropneumoniae* 4074, re-annotations were performed based on the orthologous information between strains JL03 [44] and 4074 with the stringent parameters (identities ≥80% and coverage ≥90%). All the data are MIAME compliant and the raw data has been deposited in the NCBI GEO database under the number GSE25516.

### Real-time quantitative RT-PCR

RNA was extracted as described above and reverse-transcribed into cDNA using Superscript II reverse transcriptase (Invitrogen, USA). Real time quantitative RT-PCR (qRT-PCR) was performed using ABI Power SYBR Green PCR Master Mix (ABI, USA) and the 7900 HT Sequence Detection System (ABI, USA) with 50°C, 5 min; 95°C, 10 min; 40 cycles of 95°C, 15 s; 60°C, 1 min. The primers used for real-time qRT-PCR are listed in Table S1.The relative transcription level of each gene was determined by normalization to that of the *cysQ* gene which displayed no changes in the present microarray analysis using the 2^−ΔCtΔCt^ method [45].

### Cytotoxicity assay

Cytotoxicity assays were carried out as described previously [46]. Briefly, mid-log phase cell-free supernatants (CFS) of *A. pleuropneumoniae* were harvested. CFS (1 ml) diluted 10-fold in TS buffer (10 mM Tris-HCl and 0.9% NaCl, pH 7.5) supplemented with 10 mM CaCl_2_ were incubated with mono-layer SJPL cells in 24-well plates for 4.5 hours. The supernatants were then tested using the CytoTox 96 LDH kit (Promega) according to the manufacturer's instructions. TritonX-100 (2%) and TS buffer were the positive and negative controls, respectively. Relative cytotoxicity was calculated as the percentage of the sample value normalized to the positive control. Following removal of supernatants, SJPL mono-layer cells were washed four times with phosphate buffered solution (PBS) to remove dead cells. The remaining cells were stained with crystal violet (1%) for 10 min at room temperature. Then, crystal violet was removed, stained cells were washed four times with PBS. Finally, the cells were destained with acetic acid (33%) for 10 min and the optical density at 600 nm (OD_600_) of the destaining solution was measured. The percentage of live cells remaining was calculated as the OD_600_ values of samples compared with the negative control.

### Biofilm formation detection

The detection of biofilm formation was performed using the 96-well microplate assay as previously reported [36]. Plates with the bacterial inocula were incubated from 18 h to 72 h at 37°C. Crystal violet was used to detect the quantity of biofilm. The results were determined as the ratio of OD_600_ values of biofilm/cell density [47].

### Adherence assay

The SJPL cell line was used to test the adherence ability of *A. pleuropneumoniae* strains [35]. Bacteria were cultured to mid-log phase, harvested and washed three times with DMEM and incubated with mono-layer cells cultured in six-well plates with MOIs of 100∶1 or 10∶1 for 1 to 3 hours. Non-adherent bacteria were removed by washing four times with DMEM. The number of adherent bacteria per well was determined as follows: cells with adherent bacteria were released from the wells without lysis by adding 100 µl of trypsin-EDTA (Gibco, USA) and resuspended in 900 µl TSB, then serial dilutions were plated on TSA to count the CFU of bacteria.

## Results

### Overview of microarray analysis

To investigate the *A. pleuropneumoniae* responses to catecholamines, transcriptional profiles were analyzed using microarrays to compare the Epi or NE treated and untreated *A. pleuropneumoniae*. There was no difference in growth characteristics between *A. pleuropneumoniae* grown with or without Epi or NE supplementation (Fig. S1). To see if the effects caused by Epi/NE could be inhibited by the adrenergic receptor antagonists, the non-selective α-adrenergic receptor antagonist phentolamine (PE) and the non-selective β-adrenergic receptor antagonist propranolol (PO) were also used in the phenotype tests. Before phenotypic investigations, PE and PO at 50 µM were also confirmed to have no effect on the growth of *A. pleuropneumoniae* under the conditions used in this study (Fig. S1). Since many *A. pleuropneumoniae* virulence genes (unpublished data) including those encoding Apx toxins can be differentially expressed according to growth phase [48], all cultures used for transcriptional profiling and phenotypic investigations were evaluated at the same growth phase and optical density to avoid the confusion between growth phase and hormone mediated regulation.

All samples for microarray study were harvested from mid-log phase (4 hours after sub-culture) at an OD_600_ = 1.435±0.065 for the control group, an OD_600_ = 1.461±0.019 for the Epi treated group and an OD_600_ = 1.545±0.115 for the NE treated group. Three biological replicates were conducted for each treatment. Genes whose expression changed more than 1.5-fold ( *P*<0.05) were designated as differentially expressed in accordance with previous studies [49], [50]. One hundred and fifty-seven genes were regulated by Epi with 57 and 100 genes being induced and repressed, respectively. NE regulated 104 genes including 60 up-regulated genes and 44 down-regulated genes. The extent of gene regulation by Epi was greater than that by NE. Among the Epi regulated genes, 51 genes were differentially expressed 2–3-fold and 15 genes were regulated more than 3-fold, whereas NE regulated 27 genes by 2–3-fold and only 1 gene more than 3-fold.

**Figure 1:**
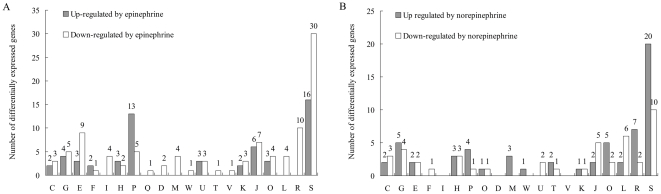
Functional classification of differentially expressed genes. (**A**). Epinephrine regulated genes. (**B**). Norepinephrine regulated genes. Gene functions are sorted according to COG categories: C: Energy production and conversion; G: Carbohydrate transport and metabolism; E: Amino acid transport and metabolism; F: Nucleotide transport and metabolism; I: Lipid transport and metabolism; H: Coenzyme transport and metabolism; P: Inorganic ion transport and metabolism; Q: Secondary metabolites biosynthesis, transport and catabolism; D: Cell cycle control, cell division; M: Cell wall/membrane/envelope biogenesis; W: Extracellular structures; U: Intracellular trafficking, secretion, and vesicular transport; T: Signal transduction mechanisms; V: Defense mechanisms; K: Transcription; J: Translation, ribosomal structure and biogenesis; O: Posttranslational modification, protein turnover, chaperones; L: Replication, recombination and repair; R: General function prediction only; S: Function unknown or not in COG classes.

According to the COG database, the proteins encoded by differentially expressed genes can be classified into 20 categories covering all aspects of metabolism, cellular processes, signaling, transcription and translation (Fig. 1). The greatest number of the regulated genes was classified as unknown or of unknown function. Only a small number of genes involved in central metabolic pathways were affected, consistent with the observation that no significant growth difference was observed following Epi or NE treatment. Genes encoding many virulence factors and regulatory proteins were identified.

Nine genes associated with virulence and regulatory roles were selected for qRT-PCR analysis. Most of the expression changes revealed by qRT-PCR were consistent with those observed by microarray analysis (Table 1). Most of the fold changes of qRT-PCR were higher than those of the results from microarray. The *ygiY* and *narQ* genes did not show significant changes in microarrays (P≥0.05), but showed significant changes in qRT-PCR analysis (P<0.05). Hence, *ygiY* and *narQ* were designated as differentially expressed genes in the following analysis.

**Table 1:** Validation of microarray results by real-time quantitative RT-PCR (qRT-PCR).

Gene name	Epinephrine		Norepinephrine	
	Fold change of qRT-PCR	Fold change of microarray	Fold change of qRT-PCR	Fold change of microarray
*apxIA*	**2.62±0.24**	**1.78**	**−1.89±0.09**	**−2.00**
*apxIIA*	−1.11±0.05	−1.35	**−2.81±0.12**	**−2.44**
*APL_0443*	**−4.80±0.03**	**−1.54**	**2.09±0.31**	**1.61**
*ygiY*	**3.59±0.60**	1.13	**−2.87±0.07**	−1.64
*gyiX*	**2.81±0.05**	**1.59**	**−2.99±0.04**	**−1.51**
*luxS*	−1.02±0.13	−1.03	**1.52±0.17**	**1.98**
*narQ*	**1.39±0.13**	2.28	**−1.46±0.13**	−1.39
*narP*	**−1.36±0.11**	**−2.29**	**1.45±0.08**	**1.90**
*pagB*	**2.92±0.21**	**2.65**	**−2.18±0.10**	**−2.08**

Fold changes with P<0.05 are shown in bold.

### Genes regulated by both Epi and NE

It was notable that the two catecholamine tested regulated different sets of genes in *A. pleuropneumoniae*. Based on the microarray and qRT-PCR results, only 18 genes were regulated by both Epi and NE. Sixteen genes were regulated oppositely by the two hormones and 2 genes were down-regulated by both hormones (Table 2). Among these genes, there were two pairs of two-component system genes, *ygiY-ygiX* (designated *qseC-qseB* in *E. coli*) and *narQ-narP*. The gene expression of several virulence factors were modulated by the two hormones. The major virulence gene *apxIA*, encoding the pore forming RTX toxin ApxIA [51], was induced by Epi but repressed by NE. The *pgaB* gene involved in PGA synthesis and crucial for biofilm formation [52] was also induced by Epi but repressed by NE. The APL_0443 gene (encoding an autotransporter adhesin [31]) and APL_1942 (encoding a Zn-dependent protease [44]) were down-regulated by Epi but up-regulated by NE. Genes encoding the tyrosine-specific transport protein P2 (*tyrP2*) and the phosphofructokinase gene (*pfkA*) involved in glycolysis were down-regulated by Epi but up-regulated by NE.

**Table 2:** Genes regulated by both epinephrine (Epi) and norepinephrine (NE).

Gene locus tag	Gene name	Description	Fold change regulated by Epi	Fold change regulated by NE
APJL_0591	*tyrP2*	tyrosine-specific transport protein	−1.84	1.62
APJL_0758	*-*	hypothetical protein	−1.98	1.62
APJL_0773	*-*	hypothetical protein	−2.28	2.07
APJL_1024	*-*	inner membrane protein	−2.54	1.86
APJL_1143	*pfkA*	phosphofructokinase	−2.84	2.62
APJL_1398	*-*	hypothetical protein	−1.75	1.60
APJL_1942	*-*	Zn-dependent protease with chaperone function	−1.58	1.56
APJL_1992	*-*	integral membrane protein	−2.12	1.54
APL_0443	*-*	autotransporter adhesin	−1.54	1.61
APL_0540	*-*	thiamine monophosphate synthase	−1.54	1.58
APJL_0059	*narP*	nitrate/nitrite response regulator protein	−2.29	1.90
APJL_1079	*ygiX*	transcriptional regulatory protein	1.59	−1.51
APJL_1080	*ygiY* a	sensor protein	1.13^a^	−1.64^a^
APJL_0489	*narQ* a	nitrate/nitrite sensor protein	2.28^a^	−1.39^a^
APJL_1969	*pgaB*	biofilm PGA synthesis lipoprotein PgaB precursor	2.65	−2.08
APP_1_029_13	*apxIA*	RTX-I toxin determinant A	1.78	−2.00
APJL_1404	*-*	ribosomal protein L32	−3.37	−2.34
APJL_1405	*-*	hypothetical protein	−4.58	−2.32

a Fold changes were not significant in microarray result but significant by qRT-PCR confirmation (P<0.05).

### Epinephrine regulated genes

**Figure 2:**
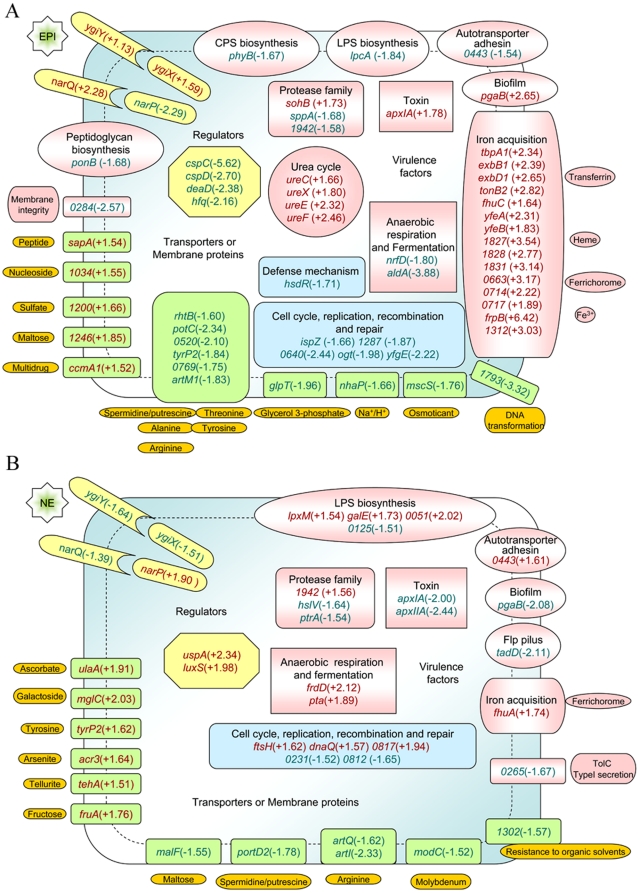
Epi and NE regulated genes involved in virulence, transport, cell cycle, DNA recombination, defense mechanism and regulation. (**A**). Epi regulated genes. (**B**). NE regulated genes. The numbers indicate the JL03 locus tag numbers of genes which have no gene name except that 0443 means APL_0443.

Epi regulated *A. pleuropneumoniae* genes functioning in metabolism and in cellular processes such as replication, recombination, transcription and translation processing (Table S2). Among these regulated genes, many known or putative virulence factors were identified. The genes associated with infection, transport and regulation were further analyzed and shown in Fig. 2A. These genes were also compared with those identified in previous transcriptional profiling studies identifying *A. pleuropneumoniae* genes regulated after growth under iron-restricted conditions [53], exposure to bronchoalveolar fluid [45], and during the acute phase of a natural infection [32]. By comparing the differentially expressed gene lists, 17, 11 and 13 Epi-regulated genes were identified to be also differentially expressed in these three studies (Table S3).

Among known virulence factors, the ApxI structural gene *apxIA* was up-regulated as mentioned above. Several genes encoding proteins involved in membrane structures were repressed. Besides the APL_0443 (an autotransporter adhesion), *phyB* (involved in CPS modification and transport), *lpcA* (involved in LPS biosynthesis), *ponB* (involved in peptidoglycan biosynthesis) were also down-regulated. APJL_0284, which has a BI-1-like family domain and plays role in membrane integrity [54], was repressed. However, the biofilm formation gene *pgaB* was up-regulated. In the protease family, in addition to APJL_1942 that was also regulated by NE, two other genes encoding proteases (*sohB* and *sppA*) were induced and repressed by Epi, respectively.

Many genes involved in metabolism but also critical for infection were affected. The genes *ureC*, *ureX*, *ureE* and *ureF*, encoding urease subunits which is involved in resistance to macrophage damage and bacterial chronic infection [27], [28], [55], were all induced by Epi. The *nrfD* gene (encoding a nitrate reductase subunit involved in anaerobic respiration) and *aldA* (encoding aldehyde dehydrogenase involved in fermentation) were down-regulated by Epi.

Several genes encoding transporters were affected by Epi. Notably, 15 genes associated with iron-acquisition and metabolism were induced, most more than 2-fold. These genes function in acquisition of different forms of iron including transferrin, heme, ferrichrome and Fe^3+^. The crucial virulence protein iron-regulated outer membrane protein B (encoded by *frpB* [56]) was induced more than 6-fold. APJL_0714 encoding the enterochelin transport protein, involved in iron-acquisition in the presence of catecholamines in some species [7], [20], was induced 2.22-fold. Besides iron-acquisition genes, others associated with the transport of different nutrients were also regulated. A putative DNA transformation gene (APJL_1793) was down-regulated more than 3-fold. A gene encoding a small-conductance mechano-sensitive channel (APJL_1923, *mscS*), involved in bacterial survival under osmolarity stress [57], was down-regulated. This regulation of transporters demonstrated that nutrition uptake and stress related exchange of substances could be regulated by Epi.

Several regulators were differentially expressed by Epi. Besides the same two-component system genes regulated by NE, two cold shock proteins encoded by *cspC* and *cspD* and a cold-shock DEAD box protein-A encoded by *deadD* were repressed by Epi. All these genes were repressed more than 2-fold. The *cspC* was repressed 5.62-fold. In addition, the host factor-I protein encoded by *hfq*, an mRNA regulatory protein [58], was repressed 2.16-fold.

### Norepinephrine regulated genes

NE regulated genes belonged to various COG functional categories (Table S4). Genes associated with infection, transport and regulatory functions were further analyzed (shown in Fig. 2B). In addition, the NE-regulated genes were compared with those differentially expressed under iron-restricted conditions [53], after exposure to bronchoalveolar fluid [45] and during the acute phase of a natural infection [32]. The results showed that only 12, 8 and 5 NE-regulated genes were also differentially expressed in these three studies (Table S5).

Among the regulated virulence genes, *apxIA*, *apxIIA* (encoding the structural gene of ApxII [59]) and APJL_0265 (encoding the membrane protein TolC involved in secretion of the Apx toxins [60]) were down-regulated. Besides the APL_0443 (encoding an autotransporter adhesin), several genes involved in membrane structures were regulated. Three genes involved in LPS biosynthesis (*lpxM*, *galE*, APJL_0051) were induced and one gene (APJL_0125) was repressed. The *pgaB* gene involved in biofilm formation, the *tadD* gene involved in Flp pilus assembly [35] and two genes (*hslV*, *ptrA*) belonging to the protease family were down-regulated.

Genes involved in *A. pleuropneumoniae* metabolism and survival during infection were also regulated. The regulation of anaerobic respiration gene expression by NE was opposite to that by Epi. The gene *frdD* (encoding a fumarate reductase subunit involved in anaerobic respiration [30]) and *pta* (encoding phosphate acetyltransferase involved in fermentation) were up-regulated. Some genes encoding transporters were affected. Only one gene involved in iron acquisition (*fhuA*) was up-regulated. Besides *fhuA*, some other inorganic ion, sugar and amino acid transport genes were induced. In contrast, APJL_1302 (encoding an ABC-type transporter involved in resistance to organic solvents) was down-regulated. These gene expression changes showed that NE exposure resulted in changes in the uptake of nutrients and stress-related exchange of substances, but the targets were different compared with that of Epi.

Six regulatory genes were affected by NE. Besides genes encoding two component regulatory systems (*ygiY-ygiX* and *narQ-narP*), the *uspA* (encoding universal stress protein A involved in stress resistance) and *luxS* (encoding autoinducer-2 production protein involved in quorum sensing) were both induced.

### Cytotoxic activity was affected in response to Epi and NE

**Figure 3:**
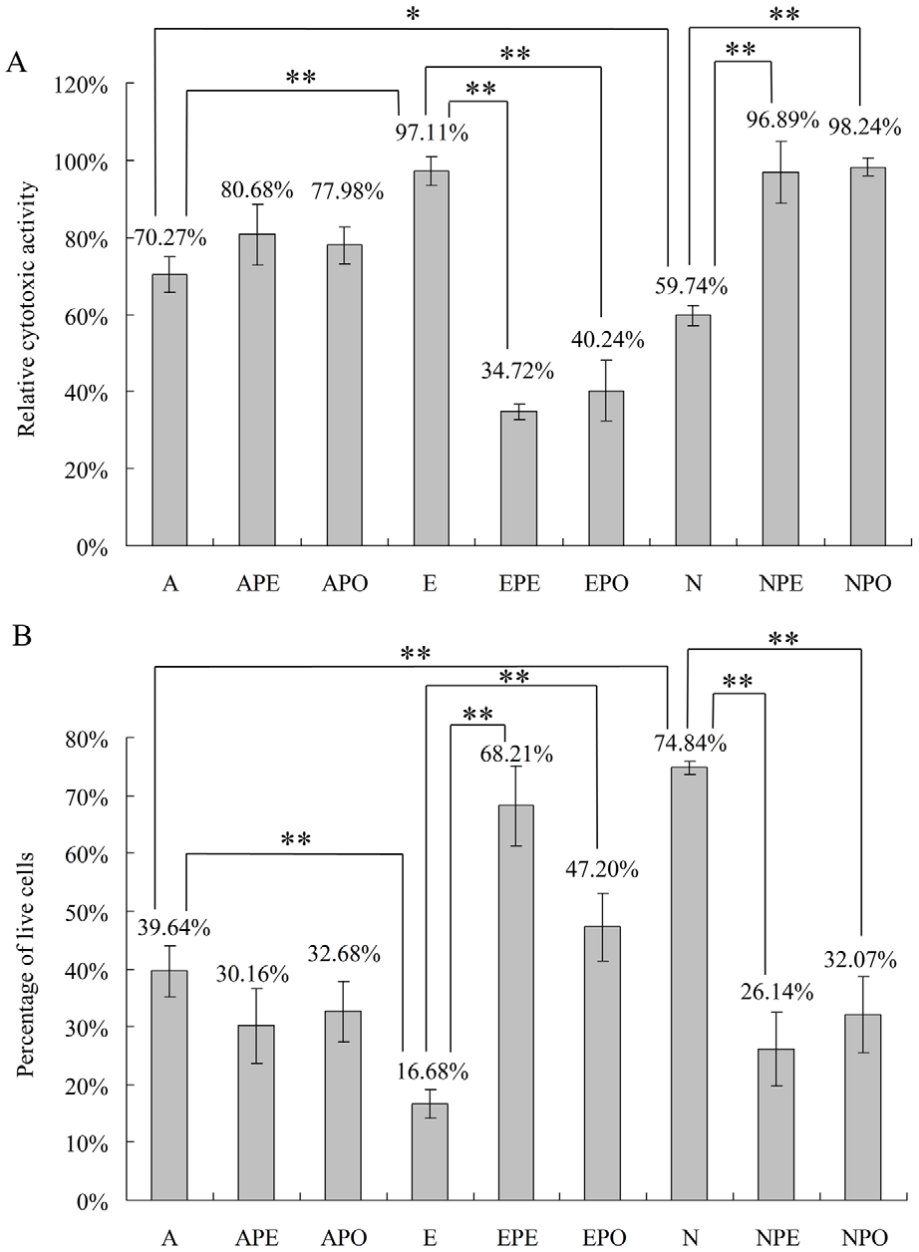
Effects of Epi and NE on *A. pleuropneumoniae* cytotoxic activities. Mid-log phase cell-free supernatants (CFS) from *A. pleuropneumoniae* (A), *A. pleuropneumoniae* supplemented respectively with 50 µM of PE (APE), PO (APO), Epi (E), Epi+PE (EPE), Epi+PO (EPO), NE (N), NE+PE (NPE), NE+PO (NPO) were tested. (**A**). Cytotoxic effects are shown as relative cytotoxic activities normalized with the positive control using 2% TritonX-100. (**B**). The percentage of live cells remaining are shown as OD_600_ values compared with the negative control using assay buffer after dyeing with crystal violet. Data are shown as means ± SD from four independent replications. The asterisk shows significant differences (one asterisk p<0.05 and two asterisks p<0.01).

Both ApxI and ApxII pore-forming exotoxins are secreted by *A. pleuropneumoniae* 4074 (serovar 1). Microarray and qRT-PCR results showed that the gene *apxIA* was up- and down-regulated by Epi and NE respectively and NE also down-regulated *apxIIA*. Hence, the effect of Epi and NE on *A. pleuropneumoniae* cytotoxic activity was evaluated using a cytotoxicity assay. For cytotoxicity detection, mid-log phase cell-free supernatants (CFS) of *A. pleuropneumoniae* with or without treatment with Epi, NE, PE, PO, Epi+PE/PO, NE+PE/PO were incubated with the SJPL cells. After incubation, cytotoxic effects were analyzed using a CytoTox 96 LDH kit and also by staining with crystal violet. Both methods showed that cytolytic activity was significantly increased by Epi but repressed by NE (*P*<0.05) (Fig. 3), consistent with the observed gene expression changes of *apxIA* and *apxIIA*. The adrenergic receptor antagonists PE and PO did not show significant influence on the cytolytic activity of bacteria not treated with Epi and NE, but could reverse the effect of Epi and NE significantly (Fig. 3). The results indicated that the antagonists could block the effects of catecholamine on cytotoxic activity. Furthermore, qRT-PCR was also performed to test the effects of adrenergic receptor antagonists on *apxIA* and *apxIIA* expression. PE and PO did not have any significant influence on *apxIA* and *apxIIA* transcription, but could inhibit the regulation on *apxIA* and *apxIIA* caused by Epi and NE (Table 3). Together, the regulation of Epi and NE on *A. pleuropneumoniae apx* genes and cytotoxic activity indicated that Epi could up-regulate *apxIA* (and hence increase cytotoxic activity), while NE could down-regulate *apxIA* and *apxIIA* (and hence decrease cytotoxic activity). All these effects could be blocked by adrenergic receptor antagonists.

**Table 3:** The effects of adrenergic receptor antagonists on Epi and NE regulation of *apxIA* and *apxIIA*.

Sample name	Fold change	
	*apxIA*	*apxIIA*
APE	1.14±0.09	1.09±0.11
APO	−1.10±0.12	−1.29±0.11
E	**2.62±0.24**	−1.11±0.05
N	**−1.89±0.09**	**−2.81±0.12**
EPE	1.24±0.15	1.10±0.05
EPO	1.10±0.03	−1.39±0.07
NPE	1.01±0.11	1.10±0.12
NPO	−1.24±0.12	−1.28±0.10

*A. pleuropneumoniae* cultured with 50 µM of PE (APE), PO (APO), Epi (E), Epi+PE (EPE), Epi+PO (EPO), NE (N), NE+PE (NPE) and NE+PO (NPO) are shown.

Fold changes with P<0.05 are shown in bold.

### Biofilm formation was not affected by Epi and NE

The biofim PGA synthesis gene *pgaB* was regulated by Epi and NE in both microarray and qRT-PCR assays. Thus, 96-well plate assays were carried out to test the effect of Epi and NE on biofilm formation under the conditions used for transcriptional profiling. The results were observed from 18 hour to 72 hours. Biofilm formation was not affected by Epi or NE at any time point (Fig. S2). In TSB medium without serum, visible biomass formed more easily, so medium without serum was also tested. The results demonstrated that although biofilm formation was enhanced in serum-free medium, neither Epi nor NE changed the biofilm formation ability of *A. pleuropneumoniae* (Fig. S2).

### Adhesion to host cells was enhanced by NE but not Epi

**Figure 4:**
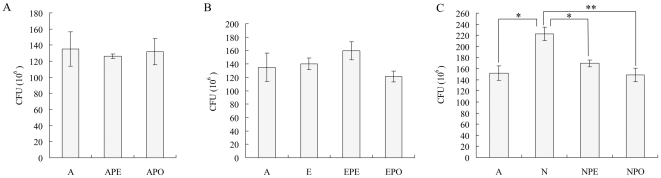
Effects of Epi and NE on *A. pleuropneumoniae* adherence abilities. *A. pleuropneumoniae* (A) were cultured respectively with 50 µM of PE (APE), PO (APO), Epi (E), Epi+PE (EPE), Epi+PO (EPO), NE (N), NE+PE (NPE) and NE+PO (NPO). Mid-log phase cultures were incubated with the SJPL cells at MOI of 100∶1 for 2 hours at 37°C. The number of adhered bacteria CFU was recorded. Data are shown as means ± SD from four independent replications. The asterisk shows significant differences (one asterisk p<0.05 and two asterisks p<0.01).

Microarray and qRT-PCR analysis revealed that the APL_0443 (encoding an autotransporter adhesin) was down-regulated by Epi but up-regulated by NE. Thus, the adhesion abilities of Epi or NE treated *A. pleuropneumoniae* from mid-log phase were evaluated using the SJPL cell line. The Epi treated bacteria did not show a difference in adherence, compared to control, at any of incubation time with MOI of 100∶1 and 10∶1, respectively. But using an MOI = 100∶1, after 2 hours' incubation, NE treated bacteria showed significantly enhanced adherence ability (*P*<0.05) (Fig. 4). The antagonists PE and PO did not affect the adherence of the bacteria untreated with Epi and NE, but could decrease the adherence of NE treated bacteria significantly (*P*<0.05) (Fig. 4). Therefore, NE but not Epi could enhance *A. pleuropneumoniae* adherence to host cells and this effect could be blocked by the adrenergic antagonists.

## Discussion

In recent years, microbial endocrinology, the intersection between microbiology and neurophysiology, has given new insights into the progress of infectious disease. Studies in this field have revealed that many bacteria can actively respond to mammalian stress hormones (catecholamines) and modulate pathogenic processes [1]. Although most investigations to date have concentrated on enteric bacteria, other species including the periodontal pathogens [61], [62] and respiratory pathogens [8], [20], [21] are also reported to respond to catecholamines. *A. pleuropneumoniae* is the respiratory pathogen of swine causing porcine contagious pleuropneumonia. In this study, the transcriptional profiles revealed that *A. pleuropneumoniae* responded to catecholamines and regulated genes involved in various cell processes including many virulence traits. This finding is consistent with the fact that stressors contributed to the infection process of *A. pleuropneumoniae* [42]. Several respiratory pathogens including *B. bronchiseptica*, *B. pertussis* [8], [20] and *M. hyopneumoniae* [21] have been reported to respond to catecholamines. These findings, together with the results in this study, suggest that respiratory pathogens can actively respond to mammalian stress hormones. Acute respiratory distress is related to high concentrations of catecholamines in plasma [39]. Animal pneumonia tends to occur after stress [38] and cold stress can lead to a decrease in pulmonary bacterial clearance in pigs [63]. Bacterial responses to catecholamines may play roles in these observations.

Because catecholamines levels can change according to stress level, tissue location and circadian rhythm, the actual catecholamine concentrations inside tissues and blood are difficult to accurately determine [64]. Phagocytes are important sources of catecholamines and phagocyte-derived catecholamines can enhance inflammatory responses [6], [65], [66]. Catecholamine levels can reach millimolar concentrations following release from phagocytes [65]. The concentration of catecholamines used in this study (50 µM) is, therefore, biologically relevant. This concentration has been used in other studies which have investigated bacterial responses to catecholamines including the respiratory pathogen *B. bronchiseptica* [8], [20]. The cattle serum used for culturing *A. pleuropneumoniae* contained less than 1 µM catecholamines as determined by ELISA (data not shown). This concentration is much lower than the supplemented catecholamines (50 µM) used in this study, therefore, the catecholamines in the serum was neglected.

Before gene expression profiling, growth ability was tested. Neither Epi nor NE affected *A. pleuropneumoniae* growth in the medium used. The growth of many bacteria [7], [8], [20], [67] can be enhanced by Epi or NE in iron-restricted conditions. In this study, iron-replete medium was used to ensure the same growth rate between hormone-treated and -untreated bacteria as was observed. Therefore, Epi and NE-induced gene expression changes did not result from growth phase variation. Although Epi and NE did not stimulate *A. pleuropneumoniae* growth in this condition, Epi still up-regulated 15 genes involved in the acquisition of various forms of iron and NE also induced one iron-acquisition gene. Hence, Epi and NE may also regulate iron acquisition for nutritional need in *A. pleuropneumoniae* as what observed in other bacteria [7], [8], [20], [67]. Notably, a gene encoding a siderophore receptor, the enterochelin transport protein (APJL_0714), was induced by Epi. Catecholamines can bind iron and, in other species, are used as pseudosiderophores to support bacterial growth [68]. The genes encoding enterobactin (also called enterochelin) receptors are also up-regulated in response to catecholamines in other species [7], [20]. Such receptors have an essential role in catecholamine-stimulated growth [7], [20]. So, the function of APJL_0714 in the *A. pleuropneumoniae* response to catecholamines for iron acquisition may be important. A greater number of iron-acquisition genes were regulated by Epi than that by NE. This is possibly because the lowest concentration of Epi and NE that could be sensed by *A. pleuropneumoniae* may be different. The effects of the two hormones on *A. pleuropneumoniae* growth in iron-restricted conditions are under investigation in our lab.

Our study showed that in *A. pleuropneumoniae*, only 18 genes were regulated by both NE and Epi. In *E. coli* O157:H7, Epi and NE regulate 938 genes and 970 genes, respectively, with only 411 being regulated by both hormones [14]. The numbers of genes regulated by the two hormones are much larger in *E. coli* than that in *A. pleuropneumoniae*, but Epi and NE also influence different sets of genes in *E. coli*. In another study, NE and dopamine but not Epi can efficiently stimulate growth of *Yersinia enterocolitica* [67]. Moreover, Epi can inhibit the growth stimulation caused by NE and dopamine [67]. Thus, the influences of Epi and NE may not always the same. The converse regulation of the same genes by Epi and NE observed in this study may be associated with the structural differences in the two hormones, e.g. the methyl group in Epi is absent in NE. Alternatively, differential gene expression may result from different responsive systems for the two hormones as s suggested by Freestone [69].

Among the genes regulated by both Epi and NE, some putative hormone receptors were discovered. Both Epi and NE are derived from tyrosine, thus the tyrosine transporter gene *tyrP2* (regulated by both hormones) might have a role in catecholamine transport. Two other genes encoding membrane proteins (APJL_1024 and APJL_1992) were also regulated by Epi and NE, but the functions of these genes are unknown. Responses to catecholamine were also possibly mediated by two-component signal transduction systems (TCSTS). In *E. coli*, there are two identified adrenergic receptors: TCSTS sensor kinases QseC and QseE [17], [18], [19]. Among the Epi and NE regulated genes in *A. pleuropneumoniae*, two pairs of TCSTS genes, the *ygiY*-*ygiX* (named *qseC*-*qseB* in *E. coli*) and the *narQ*-*narP* (regulate nitrate/nitrite respiration in *E. coli* [70]), were found. Only five TCSTS (encoded by *ygiY*-*ygiX*, *narQ*-*narP*, *phoR-phoB*, *cpxR-cpxA*, *arcB-arcA*) are identified in *A. pleuropneumoniae* genome [44]. This small number of TCSTS infers that each TCSTS may sense more than one kind of signal in the environment and modulate many behaviors. Moreover, TCSTS with known functions might play different roles in different species [71]. Therefore, *ygiY*-*ygiX* and *narQ-narP* are possible signal transduction components in *A. pleuropneumoniae* in response to catecholamines. On the other hand, the different regulators affected by Epi and NE may be involved in the respective signaling pathway in response to the two different hormones. Epi down-regulated the stress related regulators encoded by *cspC*, *cspD* and *deaD* [72] and the mRNA regulator encoded by *hfq* [58]. Cold shock proteins are stress related regulators induced with decreased temperature, but also function in normal conditions [72] such as during biofilm formation [73]. The *cspC* and *hfq* were both repressed after acute infection of *A. pleuropneumoniae* [32], suggesting that they play a role in the infection process. NE induced the gene expression of *usp*A and *luxS*. The former encodes universal stress protein A (UspA), a regulator which, in *E. coli*, responds to multiple stresses [74]. The protein LuxS (encoded by *luxS*) is involved in autoinducer 2-mediated quorum sensing in some species [75] and affects biofilm formation and virulence in *A. pleuropneumoniae* [47], [76]. *E. coli* LuxS is also related to production of AI-3, the bacterial signal that cross-talks with host Epi and NE [43], [77]. These proteins which are involved in regulatory functions may play roles in Epi and NE responsive systems of *A. pleuropneumoniae*. Whether these proteins regulate the gene expression of other *A. pleuropneumoniae* virulence factors identified in this study in response to catecholamines remains to be determined.

Since stressors can have very different effects on catecholamine secretions [78], the different *A. pleuropneumoniae* response systems may exist to allow adaptation to the different catecholamines, or a combination of more than one type of catecholamine, produced according to the physiological and immune status of the host. In addition, in a few cases, genes regulated by Epi and NE were the same as those found to be regulated under iron-restriction [53], after bronchoalveolar fluid exposure [45] and during acute infection [32]. There were only a few genes in common that were differentially regulated in the three latter studies. This suggests that *A. pleuropneumoniae* modulates its gene expression in response to different stressors using multiple regulatory mechanisms.

On the basis of the microarray and qRT-PCR results, the phenotypic effects of Epi and NE on specific virulence traits of *A. pleuropneumoniae* were tested further. The results confirmed that cytotoxic activity was enhanced by Epi but repressed by NE, paralleling the gene expression of *apxIA*. ApxI encoded by the operon *apxICABD* is strongly cytotoxic [51]. Although only the structural gene *apxIA* was significantly induced by Epi and repressed by NE more than 1.5-fold, the other genes in the operon showed the same up- and down-regulation trend by Epi and NE, respectively. Meanwhile, the genes encoding ApxIIA (which is moderately cytotoxic [51]) and the Apx toxin secretion protein TolC [60] were also down-regulated by NE, suggesting these two factors also contribute to the repression of cytotoxicity by NE. Thus, cytotoxic activity could be induced by Epi and repressed by NE through regulation of Apx toxins, which may be important to the *A. pleuropneumoniae* infection process. However, no regulator of *A. pleuropneumoniae* Apx toxins was reported. The molecular mechanism of catecholamine regulation on *apx* genes and cytotoxic activity needs further investigation.

Biofilm formation is believed to be involved in the infection of *A. pleuropneumoniae* [36]. The *pgaB* (encoding the biofilm PGA synthesis lipoprotein) was regulated by Epi and NE. In the *pga* operon, *pgaA* and *pgaC* showed similar expression trend but *pgaD* had some inconsistent up- and down-regulation trend. This may due to probe sequence or other unknown regulation of *pgaD*. The expression of H-NS and the sigma E factor known to regulate the *pga* operon [79], [80] was not significantly changed by Epi or NE, indicating that other factors regulated this operon in response to catecholamines. However, biofilm formation was not affected by either Epi or NE in the medium with or without serum. It is possible that some further regulatory mechanisms of the *pga* operon exist. Many genes have been reported to influence the biofilm formation of *A. pleuropneumoniae* [47], [80], [81], [82]. Hence, the lack of effect on biofilm formation may be the result of a number of complicated modulations.

The APL_0443 encoding autotransporter adhesin was repressed by Epi but induced by NE. However, the ability of *A. pleuropneumoniae* to adhere to host cells was induced by NE but not affected by Epi. This result may due to a complex interplay of factors including the LPS [83], [84], [85], [86], [87], type IV pilus [32], [33], [34] , Flp pilus and biofim [35] contributing to adherence of *A. pleuropneumoniae* to host cells. The effects of other adhesins could mask the differential expression of the autotransporter adhesin. For example, *pgaB* showed differential regulation by both Epi and NE compared with APL_0443. LPS biosynthesis genes were also regulated by the two hormones. The *tadD* in the *flp* pilus operon was down-regulated by NE. Investigations on the roles and relationships of these putative adhesins could explain the molecular mechanism of increased adhesion by NE but not Epi.

In summary, this study demonstrated that *A. pleuropneumoniae* could actively respond to the stress hormones Epi and NE. The two hormones regulated many genes involved in virulence traits. Only a small number of genes, including those encoding some virulence factors and potential receptors for catecholamines, were regulated by both hormones. Further tests demonstrated that cytotoxic activity was enhanced by Epi but repressed by NE consistent with changes in gene expression of the Apx exotoxins. Adhesion to host cells was enhanced by NE but not by Epi. The results in this study suggest that stress hormones can influence the *A. pleuropneumoniae* infection process and that the bacterium may use more than one responsive system for different catecholamines. Further understanding of the *A. pleuropneumoniae* response to catecholamines can be achieved by identification of both the catecholamine receptors and the signaling pathways.

## Supporting Information

Figure S1 **Growth curves of Epi, NE and/or adrenergic receptor antagonists treated** ***A. pleuropneumoniae*** **.** (**A**). Growth curves of *A. pleuropneumoniae* (A), *A. pleuropneumoniae* supplemented respectively with 50 µM of PE (APE) and PO (APO) respectively. (**B**). Growth curves of *A. pleuropneumoniae* (A), *A. pleuropneumoniae* supplemented respectively with 50 µM of Epi (E), Epi+PE (EPE), Epi+PO (EPO) respectively. (**C**). Growth curves of *A. pleuropneumoniae* (A), *A. pleuropneumoniae* supplemented respectively with 50 µM of NE (N), NE+PE (NPE), NE+PO (NPO) respectively. The arrows indicate the time of samples harvested for transcriptional profiling and phenotypic investigations. Data are shown as means ± SD from four independent replications.
(TIF)

Figure S2 **Effects of Epi and NE on** ***A. pleuropneumoniae*** **biofilm formation.** *A. pleuropneumoniae* were cultured with (+serum) and without serum (−serum) respectively for 72 hours without shaking. Epi (E) and NE (N) were added at 50 µM respectively. Biofilm formations are represented by OD_600_ values of biofilm normalized with OD_600_ values of bacteria cell densities. Data are shown as means ± SD from four independent replications.
(TIF)

Table S1 **Primers used in this study.**
(DOC)

Table S2 ***A. pleuropneumoniae*** **genes which are differentially expressed in response to epinephrine.** Genes with fold change more than 1.5 fold and p<0.05 are displayed in this table. Genes are sorted according to their function in COG classes.
(DOC)

Table S3 **Epinephrine regulated genes reported to be differentially expressed in other studies.**
(DOC)

Table S4 ***A. pleuropneumoniae*** **genes which are differentially expressed in response to norepinephrine.** Genes with fold change more than 1.5 fold and p<0.05 are displayed in this table. Genes are sorted according to their function in COG classes.
(DOC)

Table S5 **Norepinephrine regulated genes reported to be differentially expressed in other studies.**
(DOC)
