# Supplementary figures and images for: Global Effects of Catecholamines on Actinobacillus pleuropneumoniae Gene Expression
Source: PLoS One. 2012 Feb 8;7(2):e31121. doi: 10.1371/journal.pone.0031121 (PMC3275570; doi:10.1371/journal.pone.0031121)

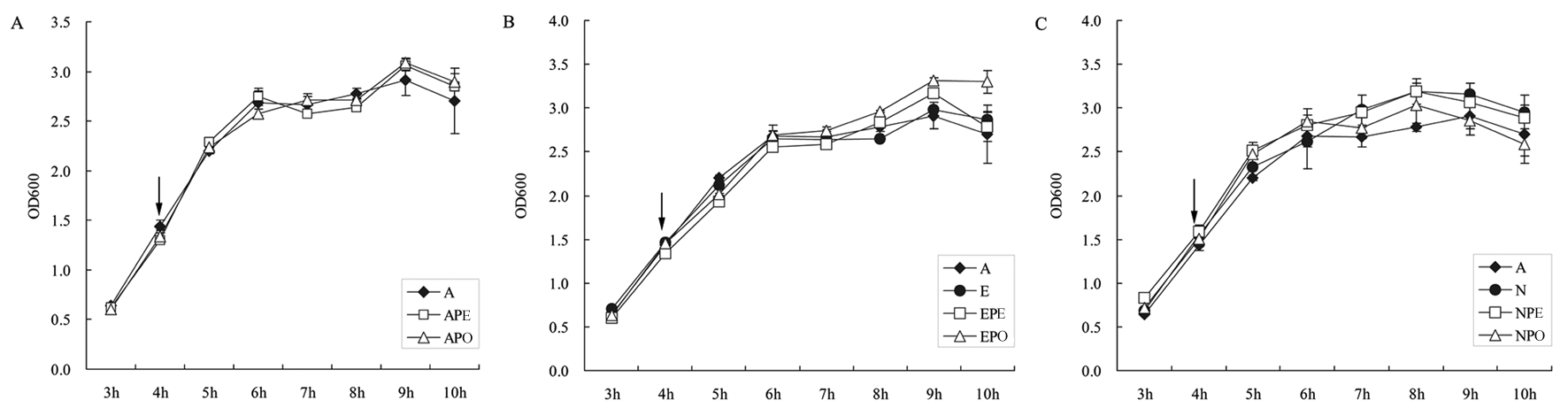

Supplement: Figure S1 — Growth curves of Epi, NE and/or adrenergic receptor antagonists treated A. pleuropneumoniae . (A). Growth curves of A. pleuropneumoniae (A), A. pleuropneumoniae supplemented respectively with 50 µM of PE (APE) and PO (APO) respectively. (B). Growth curves of A. pleuropneumoniae (A), A. pleuropneumoniae supplemented respectively with 50 µM of Epi (E), Epi+PE (EPE), Epi+PO (EPO) respectively. (C). Growth curves of A. pleuropneumoniae (A), A. pleuropneumoniae supplemented respectively with 50 µM of NE (N), NE+PE (NPE), NE+PO (NPO) respectively. The arrows indicate the time of samples harvested for transcriptional profiling and phenotypic investigations. Data are shown as means ± SD from four independent replications. (TIF) [file pone.0031121.s001.tif]

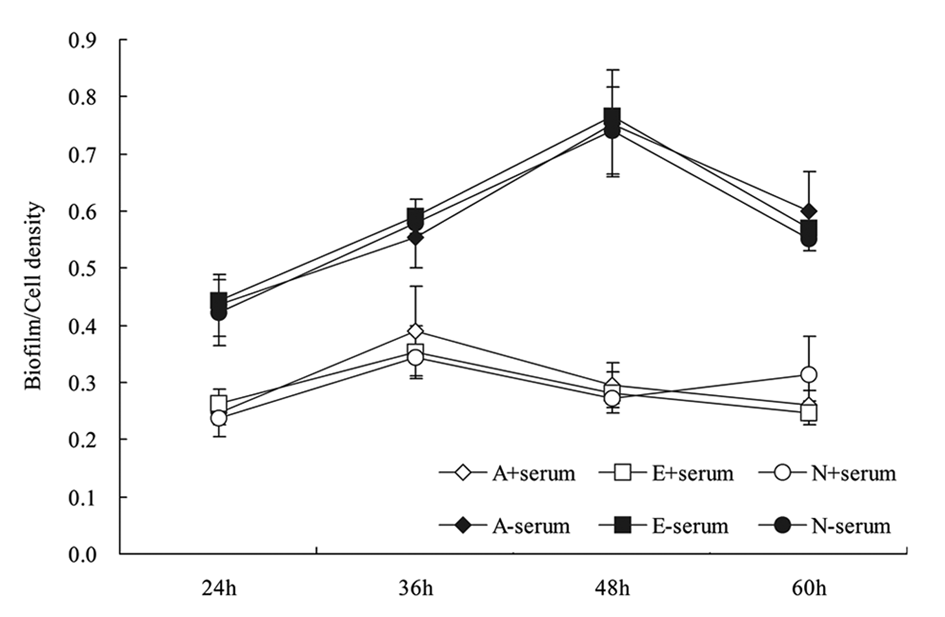

Supplement: Figure S2 — Effects of Epi and NE on A. pleuropneumoniae biofilm formation. A. pleuropneumoniae were cultured with (+serum) and without serum (−serum) respectively for 72 hours without shaking. Epi (E) and NE (N) were added at 50 µM respectively. Biofilm formations are represented by OD600 values of biofilm normalized with OD600 values of bacteria cell densities. Data are shown as means ± SD from four independent replications. (TIF) [file pone.0031121.s002.tif]
