# Supplementary material for: Global Effects of Catecholamines on Actinobacillus pleuropneumoniae Gene Expression
Source: PLoS One. 2012 Feb 8;7(2):e31121. doi: 10.1371/journal.pone.0031121 (PMC3275570; doi:10.1371/journal.pone.0031121)
Supplement: Table S1 — Primers used in this study. (DOC) [file pone.0031121.s003.doc]

**Table S**1. Primers used in this study.

| Gene name | Primers |
| --- | --- |
| *ygiY* | GGGCGGGACGGACAAT |
|  | TGTTCGATTAAGCGGACGTTT |
| *ygiX* | CAAGGGATACGTTAGACGAAAGAATA |
|  | AATGCAAACGGCTTACAGAGATAA |
| *narP* | CGCCGAGTTGGTCGAAAATA |
|  | TGGCGTACGCTGTCACTTAAGA |
| *narQ* | CCGACAAGTGAAAAACGAAGATT |
|  | CGGACGAAAGAAGACCGAGTT |
| *pgaB* | GCAAAGCGCCGCAAATTA |
|  | CGGCTTCTTTTGCCAATTTAA |
| *APL_0443* | TTGCAGCAGGTGACGTGAA |
|  | TCGCTGACCGCGTATAATTG |
| *apxIA* | CGGCTATTGCGGGTATGG |
|  | CACTGACGTCCTCACCGTTTC |
| *apxIIA* | GGTCAAGGAAATGGAGTTCAAGAT |
|  | AAAGCTAGTTTTTGCAATGTCCAA |
| *luxS* | CCGTACCGGTTTCTATATGTCGTT |
|  | CCACGCCGAAACCACATC |
| *cysQ* | CGATTTTAGCGAAGACTTATTTTGC |
|  | AATTTCGCCGTTTTCTTGCA |
