# Supplementary material for: Global Effects of Catecholamines on Actinobacillus pleuropneumoniae Gene Expression
Source: PLoS One. 2012 Feb 8;7(2):e31121. doi: 10.1371/journal.pone.0031121 (PMC3275570; doi:10.1371/journal.pone.0031121)
Supplement: Table S2 — A. pleuropneumoniae genes which are differentially expressed in response to epinephrine. Genes with fold change more than 1.5 fold and p<0.05 are displayed in this table. Genes are sorted according to their function in COG classes. (DOC) [file pone.0031121.s004.doc]

**Table S2. *A. pleuropneumoniae* genes which are differentially expressed** in response to epinephrine.

| Gene locus tag | Gene name | Description | Fold change | P-value |
| --- | --- | --- | --- | --- |
| Up-regulated by epinephrine | | |  |  |
| Energy production and conversion | | |  |  |
| APJL_1830 | *-* | plastocyanin | 3.76 | 0.0354 |
| APJL_1834 | *torA* | trimethylamine-n-oxide reductase precursor | 1.77 | 0.0083 |
| Carbohydrate transport and metabolism | | |  |  |
| APJL_1037 | *rbsK1* | sugar kinase | 1.54 | 0.0053 |
| APJL_1038 | *uxaC* | glucuronate isomerase | 1.61 | 0.0385 |
| APJL_1414 | *manA* | mannose-6-phosphate isomerase | 1.77 | 0.0370 |
| APJL_1969 | *pgaB* | biofilm PGA synthesis lipoprotein PgaB precursor | 2.65 | 0.0182 |
| Amino acid transport and metabolism | | |  |  |
| APJL_0802 | *sapA* | peptide transport periplasmic protein | 1.54 | 0.0156 |
| APJL_1649 | *ureC* | urease alpha subunit | 1.66 | 0.0279 |
| APJL_2024 | *ddc* | L-2,4-diaminobutyrate decarboxylase | 1.87 | 0.0300 |
| Nucleotide transport and metabolism | | |  |  |
| APJL_1033 | *deoC* | deoxyribose-phosphate aldolase | 1.77 | 0.0013 |
| APJL_1034 | *-* | nucleoside permease | 1.55 | 0.0025 |
| Coenzyme transport and metabolism | | |  |  |
| APJL_0691 | *moaE* | molybdopterin converting factor subunit 2 | 3.09 | 0.0174 |
| APJL_1096 | *metK* | S-adenosylmethionine synthase | 2.21 | 0.0084 |
| APJL_2063 | *fhuC* | ABC-type cobalamin/Fe3+-siderophores transportsystems, ATPase components | 1.64 | 0.0101 |
| Inorganic ion transport and metabolism | | |  |  |
| APJL_0280 | *yfeB* | iron (chelated) transporter, ATP-binding protein | 1.83 | 0.0479 |
| APL_0272 | *yfeA* | iron (chelated) ABC transporter, periplasmic-binding protein | 2.31 | 0.0075 |
| APJL_0286 | *frpB* | iron-regulated outer membrane protein | 6.42 | 0.0205 |
| APJL_0663 | *-* | lipoprotein precursor | 3.17 | 0.0412 |
| APJL_0714 | *-* | ABC-type enterochelin transport system,periplasmic component | 2.22 | 0.0043 |
| APJL_0717 | *-* | iron(III) transport system ATP-binding protein | 1.89 | 0.0206 |
| APJL_1200 | *-* | possible integral membrane sulfate transportor | 1.66 | 0.0478 |
| APJL_1312 | *-* | iron-regulated outer membrane protein | 3.03 | 0.0448 |
| APJL_1597 | *tbpA1* | transferrin-binding protein 1 precursor | 2.34 | 0.0371 |
| APJL_1827 | *-* | ABC-type Fe3+-hydroxamate transport system,periplasmic component | 3.54 | 0.0114 |
| APJL_1831 | *-* | ABC-type Fe3+-hydroxamate transport system,periplasmic component | 3.14 | 0.0141 |
| APP_1_008_55 | *-* | COG1629: Outer membrane receptor proteins, mostly Fe transport | 1.91 | 0.0346 |
| APP_1_025_24 | *-* | COG1629: Outer membrane receptor proteins, mostly Fe transport | 2.36 | 0.0417 |
| Intracellular trafficking, secretion, and vesicular transport | | |  |  |
| APJL_0009 | *sohB* | serine protease | 1.73 | 0.0188 |
| APJL_1599 | *exbD1* | biopolymer transport ExbD protein | 2.65 | 0.0323 |
| APJL_1600 | *exbB1* | biopolymer transport ExbB protein | 2.39 | 0.0410 |
| Transcription | |  |  |  |
| APJL_1079 | *ygiX* | transcriptional regulatory protein | 1.59 | 0.0217 |
| APJL_1699 | *rbsR* | ribose operon repressor | 3.50 | 0.0339 |
| Translation, ribosomal structure and biogenesis | | |  |  |
| APJL_0220 | *thrS* | threonyl-tRNA synthetase | 1.79 | 0.0443 |
| APJL_0603 | *pheT* | phenylalanyl-tRNA synthetase, beta subunit | 1.57 | 0.0251 |
| APJL_1179 | *-* | probable pseudouridine synthase | 1.47 | 0.0315 |
| APJL_1755 | *rplL* | 50S ribosomal protein L7/L12 | 1.52 | 0.0435 |
| APJL_1817 | *rpsM* | 30S ribosomal protein S13 | 2.02 | 0.0417 |
| APJL_1921 | *rluC* | ribosomal large subunit pseudouridine synthase C | 1.88 | 0.0468 |
| Posttranslational modification, protein turnover, chaperones | | |  |  |
| APJL_1646 | *ureF* | urease accessory protein UreF | 2.46 | 0.0152 |
| APJL_1647 | *ureE* | urease accessory protein UreE | 2.32 | 0.0355 |
| APJL_1648 | *ureX* | UreX | 1.80 | 0.0333 |
| Function unknown or not in COG | |  |  |  |
| APJL_0076 | *tonB2* | TonB energy transducing protein | 2.82 | 0.0149 |
| APJL_0434 | *-* | outer membrane protein | 1.54 | 0.0200 |
| APJL_0722 | *-* | hypothetical protein | 1.63 | 0.0232 |
| APJL_0835 | *-* | hypothetical protein | 1.84 | 0.0052 |
| APJL_0846 | *ccmA1* | ABC-type multidrug transport system, ATPase component | 1.52 | 0.0063 |
| APJL_1246 | *-* | maltose operon periplasmic protein | 1.85 | 0.0281 |
| APJL_1430 | *oapA* | opacity associated protein A | 1.71 | 0.0188 |
| APJL_1628 | *-* | hypothetical protein | 1.55 | 0.0441 |
| APJL_1828 | *-* | Fe(III) dicitrate ABC transporter, permease | 2.77 | 0.0247 |
| APJL_1832 | *-* | hypothetical protein | 2.00 | 0.0046 |
| APJL_1928 | *-* | hypothetical protein | 1.66 | 0.0268 |
| APJL_1929 | *-* | hypothetical protein | 1.55 | 0.0312 |
| APJL_2081 | *-* | hypothetical protein | 2.17 | 0.0254 |
| APJL_2082 | *-* | thiol-disulfide isomerase and thioredoxins | 1.52 | 0.0283 |
| APP_1_029_13 | *apxIA* | RTX-I toxin determinant A | 1.78 | 0.0382 |
| APL_0500 | *-* | hypothetical protein | 1.73 | 0.0205 |
| Down-regulated by epinephrine | | |  |  |
| Energy production and conversion | | |  |  |
| APJL_1591 | *mioC* | MioC protein | -3.00 | 0.0195 |
| APJL_1686 | *atpI* | ATP synthase protein I | -1.56 | 0.0094 |
| APJL_2061 | *aldA* | putative aldehyde dehydrogenase | -3.88 | 0.0291 |
| Carbohydrate transport and metabolism | | |  |  |
| APJL_0395 | *glpT* | glycerol 3-phosphate transporter | -1.96 | 0.0483 |
| APJL_0804 | *lpcA* | phosphoheptose isomerase | -1.84 | 0.0466 |
| APJL_1143 | *pfkA* | phosphofructokinase | -2.84 | 0.0372 |
| APJL_1954 | *xylB2* | sugar (pentulose and hexulose) kinase | -1.90 | 0.0191 |
| APP_1_032_1 | *-* | COG0036: Pentose-5-phosphate-3-epimerase | -2.10 | 0.0327 |
| Amino acid transport and metabolism | | |  |  |
| APJL_0205 | *rhtB* | threonine efflux protein | -1.60 | 0.0161 |
| APJL_0292 | *potC* | spermidine/putrescine transport system permease protein | -2.34 | 0.0433 |
| APJL_0452 | *gdhA* | glutamate dehydrogenase | -2.60 | 0.0215 |
| APJL_0520 | *-* | putative sodium/alanine symporter | -2.10 | 0.0385 |
| APJL_0591 | *tyrP2* | tyrosine-specific transport protein | -1.84 | 0.0302 |
| APJL_0652 | *aspC* | aspartate aminotransferase | -1.93 | 0.0091 |
| APJL_0769 | *-* | putative symporter | -1.75 | 0.0266 |
| APJL_1369 | *artM1* | arginine transport system permease protein | -1.83 | 0.0457 |
| APJL_1876 | *asnA* | asparagine synthetase A | -2.30 | 0.0010 |
| Nucleotide transport and metabolism | | |  |  |
| APP_1_017_24 | *-* | COG0207: Thymidylate synthase | -1.54 | 0.0383 |
| Lipid transport and metabolism | | |  |  |
| APJL_0756 | *fabI* | enoyl-[acyl-carrier-protein] reductase | -2.26 | 0.0462 |
| APJL_0807 | *ispD* | 2-C-methyl-D-erythritol 4-phosphatecytidylyltransferase | -1.65 | 0.0417 |
| APJL_0808 | *ispF* | IspF | -2.37 | 0.0294 |
| APJL_1932 | *fabA* | 3-hydroxydecanoyl-(acyl-carrier protein) dehydratase | -2.21 | 0.0287 |
| Coenzyme transport and metabolism | | |  |  |
| APJL_1860 | *menB* | dihydroxynaphthoic acid synthase | -1.55 | 0.0119 |
| APL_0540 | *-* | thiamine monophosphate synthase | -1.54 | 0.0352 |
| Inorganic ion transport and metabolism | | |  |  |
| APJL_0103 | *nrfD* | nitrate reductase, transmembrane protein | -1.80 | 0.0476 |
| APJL_1022 | *nhaP* | Na+/H+ antiporter | -1.66 | 0.0350 |
| APJL_1129 | *-* | hypothetical protein | -1.80 | 0.0107 |
| APJL_1297 | *-* | rhodanese-related sulfurtransferase | -2.24 | 0.0378 |
| APJL_1407 | *-* | hypothetical protein | -2.58 | 0.0049 |
| Secondary metabolites biosynthesis, transport and catabolism | | |  |  |
| APJL_1855 | *acpP* | acyl carrier protein | -2.56 | 0.0268 |
| Cell cycle control, cell division | | |  |  |
| APJL_0991 | *ispZ* | probable intracellular septation protein | -1.66 | 0.0462 |
| APJL_1287 | *-* | antitoxin of toxin-antitoxin stability system | -1.84 | 0.0463 |
| Cell wall/membrane/envelope biogenesis | | |  |  |
| APJL_1728 | *phyB* | PhyB, capsule biosynthetic locus protein | -1.67 | 0.0357 |
| APJL_1859 | *ponB* | penicillin-binding protein 1B | -1.68 | 0.0405 |
| APJL_1923 | *-* | small-conductance mechanosensitive channel | -1.76 | 0.0387 |
| APP_1_020_4 | *-* | COG1043: Acyl-[acyl carrier protein]--UDP-N-acetylglucosamine O-acyltransferase | -1.55 | 0.0301 |
| Extracellular structures | |  |  |  |
| APL_0443 | *-* | autotransporter adhesin | -1.54 | 0.0321 |
| Intracellular trafficking, secretion, and vesicular transport | | |  |  |
| APJL_0316 | *tolB* | colicin tolerance protein | -4.50 | 0.0346 |
| APJL_1020 | *-* | probable membrane protein | -1.55 | 0.0441 |
| APJL_1279 | *sppA* | protease IV, signal peptide peptidase | -1.68 | 0.0403 |
| Signal transduction mechanisms | | |  |  |
| APJL_0339 | *-* | PhoH-like ATP-binding protein | -1.81 | 0.0327 |
| Defense mechanisms | |  |  |  |
| APJL_0304 | *hsdR* | type I site-specific deoxyribonuclease | -1.71 | 0.0034 |
| Transcription | |  |  |  |
| APJL_0059 | *narP* | nitrate/nitrite response regulator protein | -2.29 | 0.0453 |
| APJL_0119 | *cspC* | cold shock-like protein | -5.62 | 0.0334 |
| APJL_0726 | *cspD* | cold shock-like protein CspD | -2.70 | 0.0204 |
| Translation, ribosomal structure and biogenesis | | |  |  |
| APJL_0177 | *pcnB* | polyA polymerase | -1.60 | 0.0158 |
| APJL_0568 | *deaD* | cold-shock DEAD box protein-A | -2.38 | 0.0122 |
| APJL_0851 | *-* | probable tRNA/rRNA methyltransferase | -1.68 | 0.0367 |
| APJL_0953 | *-* | putative adenine-specific methylase | -1.51 | 0.0139 |
| APJL_1404 | *-* | ribosomal protein L32 | -3.37 | 0.0476 |
| APJL_1743 | *-* | peptide deformylase | -2.43 | 0.0367 |
| APJL_2019 | *-* | ribosomal protein L33 | -2.37 | 0.0477 |
| Posttranslational modification, protein turnover, chaperones | | |  |  |
| APJL_0036 | *-* | putative protein-S-isoprenylcysteine methyltransferase | -1.75 | 0.0125 |
| APJL_0908 | *fdhE* | formate dehydrogenase formation protein | -2.26 | 0.0429 |
| APJL_1042 | *-* | peptide methionine sulfoxide reductase | -2.59 | 0.0498 |
| APJL_1942 | *-* | Zn-dependent protease with chaperone function | -1.58 | 0.0120 |
| Replication, recombination and repair | | |  |  |
| APJL_0640 | *-* | hypothetical protein | -2.44 | 0.0473 |
| APJL_0944 | *ogt* | methylated-DNA--protein-cysteine S-methyltransferase | -1.98 | 0.0355 |
| APJL_1391 | *yfgE* | ATPase involved in DNA replication initiation | -2.22 | 0.0279 |
| APP_1_026_31 | *-* | COG0593: ATPase involved in DNA replication initiation | -2.72 | 0.0349 |
| General function prediction only | | |  |  |
| APJL_0174 | *-* | cytosine deaminase and related metal-dependent hydrolase | -2.59 | 0.0299 |
| APJL_0284 | *-* | probable transport protein | -2.57 | 0.0444 |
| APJL_0374 | *-* | component of anaerobic dehydrogenase | -1.81 | 0.0232 |
| APJL_0449 | *cvpA* | colicin V production protein | -1.77 | 0.0109 |
| APJL_0569 | *nlpI* | lipoprotein | -2.27 | 0.0483 |
| APJL_1367 | *hpaC* | 4-hydroxyphenylacetic acid hydroxylase putative coupling protein | -2.41 | 0.0276 |
| APJL_1405 | *-* | hypothetical protein | -4.58 | 0.0192 |
| APJL_2008 | *hfq* | host factor-I protein Hfq | -2.16 | 0.0308 |
| APJL_2011 | *-* | predicted pyrophosphatase | -2.45 | 0.0425 |
| APJL_2026 | *-* | inner membrane protein | -1.86 | 0.0448 |
| Function unknown or not in COG | |  |  |  |
| APJL_0098 | *ilvM* | acetolactate synthase isozyme II small subunit | -1.78 | 0.0398 |
| APJL_0111 | *-* | hypothetical protein | -1.76 | 0.0325 |
| APJL_0181 | *-* | hypothetical protein | -2.03 | 0.0471 |
| APJL_0468 | *sanA* | SanA protein | -1.58 | 0.0310 |
| APJL_0471 | *-* | hypothetical protein | -1.73 | 0.0084 |
| APJL_0494 | *-* | hypothetical protein | -1.93 | 0.0440 |
| APJL_0533 |  |  | -2.37 | 0.0311 |
| APJL_0725 | *-* | hypothetical protein | -2.39 | 0.0249 |
| APJL_0758 | *-* | hypothetical protein | -1.98 | 0.0252 |
| APJL_0773 | *-* | hypothetical protein | -2.28 | 0.0166 |
| APJL_0992 | *-* | hypothetical protein | -1.59 | 0.0074 |
| APJL_1024 | *-* | inner membrane protein | -2.54 | 0.0211 |
| APJL_1047 | *-* | hypothetical protein | -1.60 | 0.0022 |
| APJL_1142 | *-* | hypothetical protein | -1.83 | 0.0297 |
| APJL_1243 | *-* | hypothetical protein | -2.04 | 0.0223 |
| APJL_1289 | *-* | hypothetical protein | -1.64 | 0.0252 |
| APJL_1378 | *-* | hypothetical protein | -1.89 | 0.0396 |
| APJL_1398 | *-* | hypothetical protein | -1.75 | 0.0295 |
| APJL_1447 | *-* | hypothetical protein | -1.55 | 0.0124 |
| APJL_1470 | *-* | hypothetical protein | -1.71 | 0.0325 |
| APJL_1602 | *-* | predicted membrane protein | -2.33 | 0.0487 |
| APJL_1666 | *-* | hypothetical protein | -1.54 | 0.0358 |
| APJL_1729 | *-* | hypothetical protein | -1.87 | 0.0103 |
| APJL_1793 | *-* | possible DNA transformation protein | -3.32 | 0.0451 |
| APJL_1992 | *-* | integral membrane protein | -2.12 | 0.0122 |
| APJL_2020 | *-* | hypothetical protein | -1.58 | 0.0376 |
| APJL_2022 | *-* | hypothetical protein | -1.60 | 0.0229 |
| APL_1633 | *-* | hypothetical protein | -1.57 | 0.0340 |
| APP_1_010_1 | *-* | COG0494: NTP pyrophosphohydrolases including oxidative damage repair enzymes | -1.67 | 0.0400 |
| APP_1_030_12 | *-* | hypothetical protein | -1.70 | 0.0203 |

Genes with fold change more than 1.5 fold and p < 0.05 are displayed in this table.

Genes are sorted according to their function in COG classes.
