# Supplementary material for: Global Effects of Catecholamines on Actinobacillus pleuropneumoniae Gene Expression
Source: PLoS One. 2012 Feb 8;7(2):e31121. doi: 10.1371/journal.pone.0031121 (PMC3275570; doi:10.1371/journal.pone.0031121)
Supplement: Table S3 — Epinephrine regulated genes reported to be differentially expressed in other studies. (DOC) [file pone.0031121.s005.doc]

**Table S3. Epinephrine regulated genes reported to be differentially expressed in other studies.**

| Gene locus_tag | Gene name | Description | Fold change (Epi) | Reference locus_tag | Reference ID | Fold change (Reference) |
| --- | --- | --- | --- | --- | --- | --- |
| Differentially expressed genes under iron-restricted condition [1] | | | | | | |
| APJL_0076 | *tonB2* | TonB energy transducing protein | 2.82 | APL_0076 | ap0082 | 1.57 |
| APJL_0280 | *yfeB* | iron (chelated) transporter, ATP-binding protein | 1.83 | APL_0271 | ap0294 | 2.98 |
| APJL_0316 | *tolB* | colicin tolerance protein | -4.50 | APL_0303 | ap0333 | -1.29 |
| APJL_0395 | *glpT* | glycerol 3-phosphate transporter | -1.96 | APL_0377 | ap0416 | -3.71 |
| APJL_0471 | *-* | hypothetical protein | -1.73 | APL_0444 | ap0497 | -2.27 |
| APJL_0717 | *-* | iron(III) transport system ATP-binding protein | 1.89 | APL_0717 | ap0801 | 1.49 |
| APJL_0908 | *fdhE* | formate dehydrogenase formation protein | -2.26 | APL_0896 | ap1000 | -1.62 |
| APJL_1143 | *pfkA* | phosphofructokinase | -2.84 | APL_1124 | ap1255 | -2.20 |
| APJL_1297 | *-* | rhodanese-related sulfurtransferase | -2.24 | APL_1285 | ap1437 | 1.58 |
| APJL_1312 | *-* | iron-regulated outer membrane protein | 3.03 | APL_1299 | ap1453 | 3.09 |
| APJL_1647 | *ureE* | urease accessory protein UreE | 2.32 | APL_1614 | ap1785 | -1.22 |
| APJL_1649 | *ureC* | urease alpha subunit | 1.66 | APL_1616 | ap1787 | -1.45 |
| APJL_1743 | *-* | peptide deformylase | -2.43 | APL_1711 | ap1887 | 1.64 |
| APJL_1827 | *-* | ABC-type Fe3+-hydroxamate transport system,periplasmic component | 3.54 | APL_1791 | ap1972 | -1.61 |
| APJL_1834 | *torA* | trimethylamine-n-oxide reductase precursor | 1.77 | APL_1798 | ap1979 | -1.70 |
| APJL_1860 | *menB* | dihydroxynaphthoic acid synthase | -1.55 | APL_1824 | ap2005 | -1.39 |
| APJL_1923 | *-* | small-conductance mechanosensitive channel | -1.76 | APL_1880 | ap2065 | -1.37 |
| Differentially expressed genes exposed to bronchoalveolar fluid [2] | | | | | | |
| APJL_0286 | *frpB* | iron-regulated outer membrane protein | 6.42 | APL_0276 | - | 2.55 |
| APJL_0568 | *deaD* | cold-shock DEAD box protein-A | -2.38 | APL_0575 | - | 1.88 |
| APJL_0569 | *nlpI* | lipoprotein | -2.27 | APL_0576 | - | 1.46 |
| APJL_0663 | *-* | lipoprotein precursor | 3.17 | APL_0668 | - | 8.01 |
| APJL_0717 | *-* | iron(III) transport system ATP-binding protein | 1.89 | APL_0717 | - | 2.38 |
| APJL_1024 | *-* | inner membrane protein | -2.54 | APL_1006 | - | 2.32 |
| APJL_1033 | *deoC* | deoxyribose-phosphate aldolase | 1.77 | APL_1015 | - | 2.25 |
| APJL_1378 | *-* | hypothetical protein | -1.89 | APL_1360 | - | 2.48 |
| APJL_1447 | *-* | hypothetical protein | -1.55 | APL_1415 | - | -1.86 |
| APJL_1599 | *exbD1* | biopolymer transport ExbD protein | 2.65 | APL_1569 | - | 1.75 |
| APJL_1755 | *rplL* | 50S ribosomal protein L7/L12 | 1.52 | APL_1721 | - | 1.87 |
| Differentially expressed genes during the acute phase of a natural infection [3] | | | | | | |
| APJL_0009 | *sohB* | serine protease | 1.73 | APL_0008 | - | 5.39 |
| APJL_0119 | *cspC* | cold shock-like protein | -5.62 | APL_0118 | - | -4.41 |
| APJL_0292 | *potC* | spermidine/putrescine transport system permease protein | -2.34 | APL_0282 | - | -2.09 |
| APJL_0316 | *tolB* | colicin tolerance protein | -4.50 | APL_0303 | - | 2.88 |
| APJL_0569 | *nlpI* | lipoprotein | -2.27 | APL_0576 | - | -3.49 |
| APJL_0663 | *-* | lipoprotein precursor | 3.17 | APL_0668 | - | 2.42 |
| APJL_0756 | *fabI* | enoyl-[acyl-carrier-protein] reductase | -2.26 | APL_0755 | - | -1.95 |
| APJL_1020 | *-* | probable membrane protein | -1.55 | APL_1002 | - | 1.92 |
| APJL_1037 | *rbsK1* | sugar kinase | 1.54 | APL_1019 | - | 2.29 |
| APJL_1246 | *-* | maltose operon periplasmic protein | 1.85 | APL_1234 | - | 3.38 |
| APJL_1923 | *-* | small-conductance mechanosensitive channel | -1.76 | APL_1880 | - | -1.93 |
| APJL_2008 | *hfq* | host factor-I protein Hfq | -2.16 | APL_1961 | - | -2.18 |
| APJL_2061 | *aldA* | putative aldehyde dehydrogenase | -3.88 | APL_2011 | - | 2.22 |

References:

1. Deslandes V, Nash JH, Harel J, Coulton JW, Jacques M (2007) Transcriptional profiling of *Actinobacillus pleuropneumoniae* under iron-restricted conditions. BMC Genomics 8: 72.

2. Lone AG, Deslandes V, Nash JH, Jacques M, Macinnes JI (2009) Modulation of gene expression in *Actinobacillus pleuropneumoniae* exposed to bronchoalveolar fluid. PLoS One 4: e6139.

3. Deslandes V, Denicourt M, Girard C, Harel J, Nash JH, et al. (2010) Transcriptional profiling of *Actinobacillus pleuropneumoniae* during the acute phase of a natural infection in pigs. BMC Genomics 11: 98.
