# Supplementary material for: Global Effects of Catecholamines on Actinobacillus pleuropneumoniae Gene Expression
Source: PLoS One. 2012 Feb 8;7(2):e31121. doi: 10.1371/journal.pone.0031121 (PMC3275570; doi:10.1371/journal.pone.0031121)
Supplement: Table S4 — A. pleuropneumoniae genes which are differentially expressed in response to norepinephrine. Genes with fold change more than 1.5 fold and p<0.05 are displayed in this table. Genes are sorted according to their function in COG classes. (DOC) [file pone.0031121.s006.doc]

**Table S4. *A. pleuropneumoniae* genes which are differentially expressed** in response to norepinephrine.

| Gene locus tag | Gene name | Description | Fold change | P-value |
| --- | --- | --- | --- | --- |
| Up regulated by norepinephrine | | |  |  |
| Energy production and conversion | | |  |  |
| APJL_0166 | *rnfA* | probable Na+-translocating NADH-quinone oxidoreductase | 1.78 | 0.0240 |
| APJL_1553 | *frdD* | fumarate reductase, 13 kD hydrophobic protein | 2.12 | 0.0148 |
| Carbohydrate transport and metabolism | | |  |  |
| APJL_0235 | *gpmB* | phosphoglycerate mutase | 1.81 | 0.0331 |
| APJL_1143 | *pfkA* | phosphofructokinase | 2.62 | 0.0106 |
| APJL_1450 | *mglC* | galactoside ABC transporter, permease protein | 2.03 | 0.0261 |
| APJL_1548 | *-* | predicted nucleoside-diphosphate-sugarepimerase | 2.13 | 0.0120 |
| APJL_1594 | *xylB1* | sugar (pentulose and hexulose) kinase | 1.52 | 0.0326 |
| Amino acid transport and metabolism | | |  |  |
| APJL_0458 | *leuB* | 3-isopropylmalate dehydrogenase | 1.66 | 0.0020 |
| APJL_0591 | *tyrP2* | tyrosine-specific transport protein | 1.62 | 0.0244 |
| Coenzyme transport and metabolism | | |  |  |
| APJL_0118 | *bioB* | biotin synthetase | 1.76 | 0.0199 |
| APJL_0566 | *-* | putative 2-deoxy-scyllo-inosose synthase subunit | 1.54 | 0.0446 |
| APL_0540 | *-* | thiamine monophosphate synthase | 1.58 | 0.0164 |
| Inorganic ion transport and metabolism | | |  |  |
| APJL_1104 | *acr3* | arsenite efflux pump ACR3 | 1.64 | 0.0399 |
| APJL_1225 | *tehA* | tellurite resistance protein and related permease | 1.51 | 0.0076 |
| APJL_1881 | *cysI* | putative sulphite reductase beta subunit | 1.53 | 0.0358 |
| APJL_2066 | *fhuA* | outer membrane ferric hydroxamate receptor | 1.74 | 0.0392 |
| Secondary metabolites biosynthesis, transport and catabolism | | |  |  |
| APJL_1455 | *-* | hypothetical protein | 1.55 | 0.0375 |
| Cell wall/membrane/envelope biogenesis | | |  |  |
| APJL_0173 | *lpxM* | lipid A acyltransferase | 1.54 | 0.0139 |
| APJL_0770 | *dgkA* | diacylglycerol kinase | 1.54 | 0.0403 |
| APJL_1314 | *galE* | UDP-glucose-4-epimerase | 1.73 | 0.0009 |
| Extracellular structures | |  |  |  |
| APL_0443 | *-* | autotransporter adhesin | 1.61 | 0.0396 |
| Signal transduction mechanisms | | |  |  |
| APJL_0645 | *uspA* | universal stress protein A | 2.34 | 0.0156 |
| APJL_1229 | *luxS* | autoinducer-2 production protein | 1.98 | 0.0196 |
| Transcription | | |  |  |
| APJL_0059 | *narP* | nitrate/nitrite response regulator protein | 1.90 | 0.0328 |
| Translation, ribosomal structure and biogenesis | | |  |  |
| APJL_0767 | *queA* | S-adenosylmethionine:tRNA ribosyltransferase-isomerase | 1.55 | 0.0165 |
| APJL_1501 | *tyrS* | tyrosyl-tRNA synthetase | 1.63 | 0.0320 |
| Posttranslational modification, protein turnover, chaperones | | |  |  |
| APJL_0073 | *-* | conserved glutaredoxin-like protein | 1.73 | 0.0384 |
| APJL_0589 | *ftsH* | cell division protein | 1.62 | 0.0010 |
| APJL_1507 | *-* | hybrid peroxiredoxin hyPrx5 | 1.67 | 0.0371 |
| APJL_1550 | *gst* | glutathione S-transferase | 2.05 | 0.0071 |
| APJL_1942 | *-* | Zn-dependent protease with chaperone function | 1.56 | 0.0113 |
| Replication, recombination and repair | | |  |  |
| APJL_1294 | *dnaQ* | DNA polymerase III epsilon chain | 1.57 | 0.0457 |
| APL_0817 | *-* | putative exodeoxyribonuclease VII large subunit | 1.94 | 0.0003 |
| General function prediction only | | |  |  |
| APJL_0051 | *-* | 3-deoxy-D-manno-octulosonate 8-phosphate phosphatase | 2.02 | 0.0483 |
| APJL_0312 | *-* | hypothetical protein | 1.89 | 0.0254 |
| APJL_0439 | *gloB* | probable hydroxyacylglutathione hydrolase | 1.84 | 0.0132 |
| APJL_0825 | *-* | transthyretin-like periplasmic protein | 2.59 | 0.0136 |
| APJL_1293 | *sufE* | SufE protein probably involved in Fe-S center assembly | 1.99 | 0.0264 |
| APP_1_048_1 | *-* | COG2220: Predicted Zn-dependent hydrolases of the beta-lactamase fold | 1.69 | 0.0362 |
| APP_1_048_2 | *-* | COG2220: Predicted Zn-dependent hydrolases of the beta-lactamase fold | 1.53 | 0.0499 |
| Function unknown or not in COG | | | | |
| APJL_0069 | *psiE1* | phosphate-starvation-inducible protein | 1.68 | 0.0349 |
| APJL_0359 | *fruA* | phosphotransferase system, fructose-specific IIC component | 1.76 | 0.0034 |
| APJL_0510 | *-* | hypothetical protein | 1.66 | 0.0240 |
| APJL_0635 | *pta* | phosphate acetyltransferase | 1.89 | 0.0018 |
| APJL_0724 | *-* | hypothetical protein | 1.87 | 0.0126 |
| APJL_0758 | *-* | hypothetical protein | 1.62 | 0.0400 |
| APJL_0773 | *-* | hypothetical protein | 2.07 | 0.0103 |
| APJL_1024 | *-* | inner membrane protein | 1.86 | 0.0480 |
| APJL_1310 | *-* | hypothetical protein | 1.97 | 0.0192 |
| APJL_1398 | *-* | hypothetical protein | 1.60 | 0.0304 |
| APJL_1552 | *-* | hypothetical protein | 1.67 | 0.0044 |
| APJL_1747 | *ulaA* | ascorbate-specific permease IIC component UlaA | 1.91 | 0.0395 |
| APJL_1761 | *-* | hypothetical protein | 1.96 | 0.0149 |
| APJL_1871 | *-* | possible permease | 1.66 | 0.0088 |
| APJL_1992 | *-* | integral membrane protein | 1.54 | 0.0414 |
| APJL_2027 | *-* | hypothetical protein | 1.58 | 0.0104 |
| APJL_2037 | *-* | predicted membrane protein | 2.07 | 0.0086 |
| APJL_2051 | *-* | hypothetical protein | 1.65 | 0.0033 |
| APP_1_017_17 | *-* | hypothetical protein | 2.95 | 0.0293 |
| APP_1_030_17 | *-* | hypothetical protein | 1.75 | 0.0114 |
| Down-regulated by norepinephrine | | |  |  |
| Energy production and conversion | | |  |  |
| APJL_0473 | *ykgE* | putative dehydrogenase subunit | -1.59 | 0.0176 |
| APJL_0906 | *hybA1* | formate dehydrogenase, beta subunit | -1.60 | 0.0493 |
| APJL_1214 | *nfnB* | putative NAD(P)H nitroreductase | -2.00 | 0.0443 |
| Carbohydrate transport and metabolism | | |  |  |
| APJL_0062 | *mipB* | transaldolase | -2.10 | 0.0046 |
| APJL_1250 | *malF* | ABC-type sugar transport systems, permease component | -1.55 | 0.0332 |
| APJL_1252 | *malQ* | 4-alpha-glucanotransferase | -2.07 | 0.0303 |
| APJL_1969 | *pgaB* | biofilm PGA synthesis lipoprotein PgaB precursor | -2.08 | 0.0206 |
| Amino acid transport and metabolism | | |  |  |
| APJL_0386 | *potD2* | spermidine/putrescine-binding periplasmic protein | -1.78 | 0.0464 |
| APJL_1370 | *artQ* | arginine transport system permease protein | -1.62 | 0.0493 |
| Nucleotide transport and metabolism | | |  |  |
| APJL_2068 | *purC* | phosphoribosylaminoimidazole succinocarboxamide(SAICAR) synthase | -1.66 | 0.0323 |
| Coenzyme transport and metabolism | | |  |  |
| APJL_0046 | *ribF* | riboflavin biosynthesis protein | -1.55 | 0.0398 |
| APJL_0690 | *moaD* | molybdopterin converting factor, small subunit | -1.70 | 0.0163 |
| APJL_1167 | *trpG* | anthranilate synthase component II | -1.59 | 0.0469 |
| Inorganic ion transport and metabolism | | |  |  |
| APJL_0268 | *modC* | molybdenum ABC transporter, ATP-binding protein | -1.52 | 0.0480 |
| Secondary metabolites biosynthesis, transport and catabolism | | |  |  |
| APJL_1302 | *-* | ABC-type transport system involved in resistance to organic solvents, auxiliary component | -1.57 | 0.0213 |
| Intracellular trafficking, secretion, and vesicular transport | | |  |  |
| APJL_0265 | *-* | probable outer membrane protein | -1.67 | 0.0041 |
| APJL_0542 | *tadD* | Flp pilus assembly protein | -2.11 | 0.0183 |
| Signal transduction mechanisms | | |  |  |
| APJL_1371 | *artI* | ABC-type amino acid transport system,periplasmic component | -2.33 | 0.0326 |
| Transcription |  |  |  |  |
| APJL_1079 | *ygiX* | transcriptional regulatory protein | -1.51 | 0.0107 |
| Translation, ribosomal structure and biogenesis | | |  |  |
| APJL_0560 | *tsf* | elongation factor Ts | -1.84 | 0.0264 |
| APJL_0594 | *rplM* | 50S ribosomal protein L13 | -3.02 | 0.0294 |
| APJL_1192 | *rpsF* | 30S ribosomal protein S6 | -2.23 | 0.0469 |
| APJL_1223 | *-* | hypothetical protein | -1.63 | 0.0425 |
| APJL_1404 | *-* | ribosomal protein L32 | -2.34 | 0.0219 |
| Posttranslational modification, protein turnover, chaperones | | |  |  |
| APJL_1772 | *hslV* | ATP-dependent protease | -1.64 | 0.0497 |
| APJL_1926 | *ptrA* | protease III | -1.54 | 0.0178 |
| Replication, recombination and repair | | |  |  |
| APJL_0231 | *-* | transposase | -1.52 | 0.0179 |
| APJL_0812 | *-* | exonuclease VII small subunit | -1.65 | 0.0258 |
| APP_1_007_53 | *-* | transposase | -1.60 | 0.0317 |
| APP_1_012_38 | *-* | transposase | -1.80 | 0.0074 |
| APP_1_024_17 | *-* | transposase | -1.78 | 0.0081 |
| APP_1_025_12 | *-* | transposase | -1.50 | 0.0151 |
| General function prediction only | | |  |  |
| APJL_0350 | *-* | hypothetical protein | -1.78 | 0.0183 |
| APJL_1405 | *-* | hypothetical protein | -2.32 | 0.0296 |
| Function unknown or not in COG | | | | |
| APJL_0125 | *-* | hypothetical protein | -1.51 | 0.0165 |
| APJL_0216 | *-* | hypothetical protein | -2.07 | 0.0433 |
| APJL_0227 | *-* | hypothetical protein | -1.84 | 0.0072 |
| APJL_0402 | *ribD* | riboflavin-specific deaminase | -2.29 | 0.0259 |
| APJL_0550 | *-* | hypothetical protein | -1.54 | 0.0378 |
| APJL_0628 | *-* | hypothetical protein | -2.70 | 0.0231 |
| APJL_0967 | *apxIIA* | hemolysin A | -2.44 | 0.0289 |
| APP_1_029_13 | *apxIA* | RTX-I toxin determinant A | -2.00 | 0.0308 |
| APP_1_086_1 | *-* | COG2182: Maltose-binding periplasmic proteins/domains | -2.03 | 0.0432 |
| APP_1_137_1 | *-* | COG2182: Maltose-binding periplasmic proteins/domains | -2.31 | 0.0464 |

Genes with fold change more than 1.5 fold and p < 0.05 are displayed in this table.

Genes are sorted according to their function in COG classes.
