# Supplementary material for: Global Effects of Catecholamines on Actinobacillus pleuropneumoniae Gene Expression
Source: PLoS One. 2012 Feb 8;7(2):e31121. doi: 10.1371/journal.pone.0031121 (PMC3275570; doi:10.1371/journal.pone.0031121)
Supplement: Table S5 — Norepinephrine regulated genes reported to be differentially expressed in other studies. (DOC) [file pone.0031121.s007.doc]

**Table S5. Norepinephrine** regulated genes reported to be differentially expressed in other studies.

| Gene locus_tag | Gene name | Description | Fold change (NE) | Reference locus_tag | Reference ID | Fold change (Reference) |
| --- | --- | --- | --- | --- | --- | --- |
| Differentially expressed genes under iron-restricted condition [1] | | | | | | |
| APJL_0312 | *-* | hypothetical protein | 1.89 | APL_0299 | ap0329 | 1.36 |
| APJL_0402 | *ribD* | riboflavin-specific deaminase | -2.29 | APL_0382 | ap0422 | 1.43 |
| APJL_0473 | *ykgE* | putative dehydrogenase subunit | -1.59 | APL_0446 | ap0499 | -4.38 |
| APJL_0645 | *uspA* | universal stress protein A | 2.34 | APL_0655 | ap0725 | -1.59 |
| APJL_0906 | *hybA1* | formate dehydrogenase, beta subunit | -1.60 | APL_0894 | ap0998 | -5.23 |
| APJL_1143 | *pfkA* | phosphofructokinase | 2.62 | APL_1124 | ap1255 | -2.20 |
| APJL_1370 | *artQ* | arginine transport system permease protein | -1.62 | APL_1352 | ap1507 | -1.22 |
| APJL_1548 | *-* | predicted nucleoside-diphosphate-sugarepimerase | 2.13 | APL_1522 | ap1686 | 1.84 |
| APJL_1550 | *gst* | glutathione S-transferase | 2.05 | APL_1524 | ap1688 | 1.24 |
| APJL_1594 | *xylB1* | sugar (pentulose and hexulose) kinase | 1.52 | APL_1564 | ap1733 | 3.04 |
| APJL_2037 | *-* | predicted membrane protein | 2.07 | APL_1989 | ap2182 | 1.83 |
| APJL_2051 | *-* | hypothetical protein | 1.65 | APL_2002 | ap2196 | 3.40 |
| Differentially expressed genes exposed to bronchoalveolar fluid [2] | | | | | | |
| APJL_0350 | *-* | hypothetical protein | -1.78 | APL_0334 | - | 1.59 |
| APJL_0439 | *gloB* | probable hydroxyacylglutathione hydrolase | 1.84 | APL_0415 | - | 1.86 |
| APJL_0628 | *-* | hypothetical protein | -2.70 | APL_0637 | - | 3.15 |
| APJL_1024 | *-* | inner membrane protein | 1.86 | APL_1006 | - | 2.32 |
| APJL_1104 | *acr3* | arsenite efflux pump ACR3 | 1.64 | APL_1088 | - | -2.79 |
| APJL_1250 | *malF* | ABC-type sugar transport systems, permease component | -1.55 | APL_1238 | - | 4.38 |
| APJL_1294 | *dnaQ* | DNA polymerase III epsilon chain | 1.57 | APL_1282 | - | 2.15 |
| APJL_2068 | *purC* | phosphoribosylaminoimidazole succinocarboxamide(SAICAR) synthase | -1.66 | APL_2018 | - | -3.41 |
| Differentially expressed genes during the acute phase of a natural infection [3] | | | | | | |
| APJL_0227 | *-* | hypothetical protein | -1.84 | APL_0226 | - | -2.83 |
| APJL_0635 | *pta* | phosphate acetyltransferase | 1.89 | APL_0644 | - | -2.99 |
| APJL_1250 | *malF* | ABC-type sugar transport systems, permease component | -1.55 | APL_1238 | - | 3.11 |
| APJL_1252 | *malQ* | 4-alpha-glucanotransferase | -2.07 | APL_1240 | - | 3.29 |
| APP_1_ 048_1 | *-* | COG2220: Predicted Zn-dependent hydrolases of the beta-lactamase fold | 1.69 | APL_1701 | - | 2.45 |

Reference:

1. Deslandes V, Nash JH, Harel J, Coulton JW, Jacques M (2007) Transcriptional profiling of *Actinobacillus pleuropneumoniae* under iron-restricted conditions. BMC Genomics 8: 72.

2. Lone AG, Deslandes V, Nash JH, Jacques M, Macinnes JI (2009) Modulation of gene expression in *Actinobacillus pleuropneumoniae* exposed to bronchoalveolar fluid. PLoS One 4: e6139.

3. Deslandes V, Denicourt M, Girard C, Harel J, Nash JH, et al. (2010) Transcriptional profiling of *Actinobacillus pleuropneumoniae* during the acute phase of a natural infection in pigs. BMC Genomics 11: 98.
